# Maternal health challenges experienced by adolescents; could community score cards address them? A case study of Kibuku District– Uganda

**DOI:** 10.1186/s12939-020-01267-4

**Published:** 2020-11-02

**Authors:** Rebecca R. Apolot, Moses Tetui, Evelyne B. Nyachwo, Linda Waldman, Rosemary Morgan, Christine Aanyu, Aloysius Mutebi, Suzanne N. Kiwanuka, Elizabeth Ekirapa

**Affiliations:** 1 Department of Health Policy Planning and Management, Makerere University School of Public Health, P.O.Box 7072, Kampala, Uganda; 2 Department of Epidemiology and Global Health, Umeå University, 901 87 Umeå, Sweden; 3 Institute of Development Studies, Library Road, Brighton, BN1 9RE UK; 4 Department of International Health, Johns Hopkins Bloomberg School of Public Health, 615 N. Wolfe Street, Baltimore, MD 21205 USA

**Keywords:** Maternal health, Challenges, Adolescents, Community score card

## Abstract

**Introduction:**

Approximately 34.8% of the Ugandan population is adolescents. The national teenage pregnancy rate is 25% and in Kibuku district, 17.6% of adolescents aged 12–19 years have begun child bearing. Adolescents mothers are vulnerable to many maternal health challenges including; stigma, unfriendly services and early marriages. The community score card (CSC) is a social accountability tool that can be used to point out challenges faced by the community in service delivery and utilization and ultimately address them. In this paper we aimed to document the challenges faced by adolescents during pregnancy, delivery and postnatal period and the extent to which the community score card could address these challenges.

**Methods:**

This qualitative study utilized in-depth interviews conducted in August 2018 among 15 purposively selected adolescent women who had given birth 2 years prior to the study and had attended CSC meetings. The study was conducted in six sub counties of Kibuku district where the CSC intervention was implemented. Research assistants transcribed the audio-recorded interviews verbatim, and data was analyzed manually using the framework analysis approach.

**Findings:**

This study found five major maternal health challenges faced by adolescents during pregnancy namely; psychosocial challenges, physical abuse, denial of basic human rights, unfriendly adolescent services, lack of legal and cultural protection, and lack of birth preparedness. The CSC addressed general maternal and new born health issues of the community as a whole rather than specific adolescent health related maternal health challenges.

**Conclusion:**

The maternal health challenges faced by adolescents in Kibuku have a cultural, legal, social and health service dimension. There is therefore need to look at a multi-faceted approach to holistically address them. CSCs that are targeted at the entire community are unlikely to address specific needs of vulnerable groups such as adolescents. To address the maternal health challenges of adolescents, there is need to have separate meetings with adolescents, targeted mobilization for adolescents to attend meetings and deliberate inclusion of their maternal health challenges into the CSC.

## Introduction

Globally, there are more than 1.8million adolescents aged 10–19 years [1],with approximately 70% living in Low and Middle Income Countries (LMICs). Approximately 34.8% of the Ugandan population and 26.8% of Kibuku district population are adolescents. The national teenage pregnancy rate is 25% and, in Kibuku district, 17.6% of adolescents aged 12–19 years have begun child bearing [2]. Unprotected sex does not only lead to early child bearing but also to unplanned pregnancies, unsafe and illegal abortions and its complications including death, sexually transmitted infections, birth-related complications like fistula and dropping out of school [3]. Oftentimes adolescents do not disclose their reproductive health challenges to their parents or guardians due to fear and concerns about how communities will treat them, and, as a result, they do not utilize health services [4]. This is attributed to inadequate health-related information, ignorance about health needs and rights, limited financial resources, poor attitude of health care providers, and poor attitude of community members [5].

Despite the fact that adolescents have limited information about sex and its consequences within many LMICs [6], they engage in unprotected sexual activities, leaving them vulnerable to unwanted or unplanned pregnancies [7]. In Uganda, the burden of adolescent pregnancies has persistently remained high [8, 9].

The vulnerabilities of adolescent mothers in LMICs continue to challenge efforts made to improve maternal and newborn health outcomes. These mothers are faced with a web of challenges that disproportionately predispose them to higher chances of poor health outcomes [10]. Physically, their bodies are not yet ready for birth, they are socially stigmatized, lack social support to raise their children, lack financial means of survival, have limited access to family planning services, lack an education or drop out of school and will at the same time be grappling with the psychosocial issues and changes of adolescence [11]. Additionally, evidence indicates that their newborns have a lesser chance of survival, including greater chance of being stillborn, with low birth weight, or face a higher risk of neonatal death due to a series of causes including infections [12, 13].

Globally adolescent reproductive health is influenced by several complex, interrelated factors ranging from individual (personal preference, previous experience), socio-cultural (family, peers and communities, community and cultural beliefs), [14, 15] socio-economic (education, employment, financial status),and health systems (attitude of health workers, reproductive health commodities, quality of services) [16]. Although both male and female adolescents experience reproductive health challenges, female adolescents are at a greater disadvantage [17]. Considering pregnancy, female adolescents drop out of school more frequently than their male counterparts, deaths occur among female adolescents due to birth complications and unsafe abortions, females are held responsible for pregnancy and childbirth, and they face more psychosocial challenges as a consequence of neglect from their partners [18].

Due to limited engagement of the adolescents and health workers so that the health workers better understand the challenges of pregnant adolescents, practices like women having to attend antenatal care with their male partners have been put in health facilities. Pregnant women who don’t come with their partners are served last. This has led to unintended consequences like adolescents shunning antenatal care because they are, often times, neglected and/or abandoned by their partners [19, 20]. Social accountability tools such as Community Score Card (CSC) could help to bring out the voices of adolescents in setting priorities for intervention.

In Uganda, there have been several interventions put into place that have led to a steady decline of adolescent pregnancies from 31% in 2000 to 24% in 2011 [21]. Adolescent pregnancy is a public health issue of importance because it greatly contributes to high maternal/neonatal morbidity and mortality while placing a heavier burden to the already constrained health system [12]. Limited implementation and knowledge of existing policies (National Policy Guidelines and Service Standards for Sexual and Reproductive Health and Rights) [22] are a setback on the delivery of Sexual and Reproductive health (SRH) services for adolescents in Uganda. Recent studies have shown the need for adolescent-friendly services to promote reproductive health-seeking behavior and improve the quality of adolescent SRH [23]. The government of Uganda, through the MoH, recognizes the need to improve the delivery of adolescent SRH services and to better handle adolescent issues [24]. Some of the strategies adopted include: using a multi-sectoral approach [25] where the education curriculum includes sex education, communication and information materials on adolescent SRH [26] and scaling-up the number of facilities providing adolescent-friendly SRH services [27]. Despite these interventions, there remains a need to build the capacity of health workers on how to manage adolescents. There is also a need to engage families and community members, to sensitize them on adolescents’ reproductive health needs and challenges, and to involve the legal sector and religious sects in order to handle community- and cultural-related challenges [28, 29].

There is need for more holistic interventions, such as sex education, use of contraceptives, promotion of education especially for adolescent girls (and young men) and legal and cultural protection [24, 30].

In response to community and family engagement in SRH of adolescents, Makerere University School of Public Health (MakSPH) as part of the Future health Systems (FHS) and the Research in Gender and Ethics (RinGs) Consortia implemented the Community Score card (CSC) study [31]. The Community Score Card (CSC) is a participatory social accountability tool designed and used to plan, monitor and evaluate services like health services [32]. The CSC brings the service providers and the service users of a given health service together to identify and analyze challenges to service delivery and utilization in order to find a common and shared solution to the identified challenges [33, 34].

The CSC study aimed to improve Maternal and Newborn Health (MNH) service utilization and delivery through engagement of different community, political, and technical stakeholders. The study operated in six sub counties in Kibuku district in Uganda through identification of MNH service delivery and utilization challenges and finding locally feasible solutions to the challenges. The CSC was implemented through a natural experiment, which involved discussing the MNH needs, and challenges faced by community and health workers with expectations that the challenges would be addressed in the process. The details on the Kibuku CSC intervention and the contextual factors that best facilitated implementation have been highlighted as local community and political leader involvement [35]. However the ability of the CSC as a social accountability tool to address the specific maternal health needs of the adolescents was a main concern in the study since adolescents always feel shy to discuss sexuality issues in public and in the presence of adults [36]. A CSC project evaluation in Southern Africa also pointed out that there were challenges with inclusion of marginalized groups in the CSC process. Some of the marginalized groups in maternal and newborn health projects were adolescents [37].

In this paper we explored the challenges faced by adolescents during the antenatal, delivery and postnatal period and the extent to which the community score card intervention in Kibuku district addressed those challenges.

## Methods

### Design and study area

We carried out a qualitative study in August 2018 using In-depth Interviews (IDIs) to explore the maternal health challenges faced by adolescents during pregnancy, delivery and the post-natal period. We also explored the extent to which the maternal health challenges reported by adolescents had been addressed by the community score card intervention. This study was carried out in Kibuku district, which is located in the eastern region of Uganda with a population of 202,033 people and with 52% of the population being female. People aged 10–17 years are 23% of the total population [38]. The study was carried out in six sub-counties of Kibuku district where the CSC intervention was implemented. The intervention targeted the entire population of the six sub counties.

### Study methods, selection of study participants and data collection

A total of 15 IDIs were conducted among purposively selected adolescent women from six sub-counties. The sub-county score card coordinators and the Village Health Team (VHT) coordinators of the six sub-counties where the CSC was implemented were asked to identify any adolescents who had given birth 2 years prior to the study so as to reduce recall bias and these adolescents should have participated in at least one CSC meeting. Village Health Teams refer to volunteers who are actively involved in coordinating health related issues at community level. They are involved in community mobilization for health activities, promoting hygiene and sanitation, and health education.. The CSC coordinators and VHTs were paid for coordination of the project during the intervention. The IDIs were conducted face to face within the participants’ homes using a guide with open-ended questions that explored the main themes of maternal health challenges faced by adolescents and extent to which CSC addressed those maternal health challenges.

Three experienced female research assistants who had a good working knowledge of English and Lugwere (the local language) were recruited and trained in data collection. The IDI guide was translated from English to Lugwere, then back translated and compared to ensure consistency and pre-tested in Kampala district. Written consent was received from each of the interviewees. The interviews, which lasted an average of 2 hours each, were audio recorded, and transcribed verbatim to prepare for analysis.

### Data management, analysis and ethical consideration

The data was manually analyzed using the framework analysis approach [39, 40]. The transcripts were read and a thematic framework developed on word document based on the major maternal health challenges found to be faced by adolescents during the antenatal, delivery and postnatal period to answer objective one. To answer objective two, we included the major theme of, the extent to which the CSC addressed the adolescent maternal health challenges.. Five sub themes merged from maternal health challenges major theme from the data including; psychosocial challenges, physical abuse and violation of human rights, lack of legal and cultural protection, inadequate adolescent friendly services at health facilities, lack of birth preparedness. For the theme on the extent to who the CSC address the challenges emerged three sub themes including; attendance of CSC meetings by the adolescents, participation of adolescents during the meetings, and selection of indicators to be included in the CSC. We then systematically applied this framework to each of our transcripts and sifted, charted and sorted material according to the themes. We summarized our results using text and quotes from the transcripts that elaborately illustrated meanings or key messages.

Ethical clearance was obtained from the Makerere University School of Public Health Higher Degrees Research and Ethics Committee (MakSPH HDREC) and approval from the Uganda National Council of Science and Technology (UNCST), study number SS 4323. Permission to carry out the research was further sought from the Kibuku district health office. The objectives, benefits and risks of the study were explained to the study participants and written informed consent obtained from all the participants. All data obtained were treated as confidential and anonymous identifiers were used. We restricted data access to only the investigators and the three research assistants. Investigators also actively participated in the data collection process through supervision of data collection and daily debrief meetings.

## Results

Our findings from all the 15 female adolescent mothers showed that the adolescents faced a range of specific maternal challenges, which were not addressed directly by the CSC. To provide context to the findings, we provide the adolescents’ background characteristics of our study participants including; age, level of education, marital status, daily living expenses, residence and the number of pregnancies. The average age of the adolescent mothers was 16 years with majority having attained primary education up to Primary 7. Fourteen out of fifteen adolescents had been pregnant only once and one adolescent mother had been pregnant twice. Of the 15 adolescent mothers; 6 were single, 3 were married and 6 were separated.

The results are presented in six main thematic areas derived through analysis of the interview transcripts. These include: psychosocial challenges, physical abuse and violation of human rights, lack of legal and cultural protection, inadequate adolescent-friendly services at health facilities, lack of birth preparedness and the Inclusion of adolescent specific maternal health challenges in the CSC as detailed below.

### Psychosocial challenges

The psychosocial challenges we found under this study are presented below in the categories of community related psychosocial challenges and health facility related psychosocial challenges.

#### Community related psychosocial challenges

All the interviewed adolescents faced stigma and rejection from their parents and or, communities and or partners, which made them condemn themselves. The parents and communities did not approve of adolescents getting pregnant at that early age, as they would desire that their children abstain from sex until after marriage. Partners of the adolescents often denied responsibility for pregnancy and this made the girls look like they were promiscuous since they could not introduce the father of their unborn child to their families. The communities and parents therefore used offensive and abusive language towards the pregnant adolescents. This kind of language led 13 out of 15 adolescents shunning antenatal care services or seeking such care in the last trimester and even attempts of unsafe abortions among five adolescents. Rejection by parents and partners left pregnant adolescents without sufficient emotional support that is much needed during pregnancy, delivery and postnatal period. These were emphasized in the quote below.

*“He had brought money that I use it for abortion. He told me to go and look for someone who can remove* [abort the fetus]... *He gave me 30,000/-UGX [8USD] that I use to carry out an abortion but I refused … When I refused to go by his decision of abortion, he told me that even if I give birth, he would not support me”.* (Adolescent mother 2)

#### Health facility related psychosocial challenges

***Majority of the adolescents confessed that*** the health workers in some cases used inappropriate language that showed their poor attitude towards pregnant adolescents. Some health workers would use words condemning adolescents for getting pregnant, this made them feel belittled and have self-condemnation. The inappropriate language led to some adolescents shunning seeking of health services like antenatal, delivery and post-natal care. In the quotes below, adolescents talked about how the attitude of health workers led to their alienation in health facilities, internalization of guilt and feeling that they were too young thus they separated themselves from older mothers during antenatal care visits at the health facility.

*“The only problem I used to have while attending Antenatal care (ANC) was fear to sit in a queue with older mothers that I don't know; that I have never met before in my life. So what I could do, after handing in my book for registration, I would get out of the ANC room and sit outside until they [the health workers] call my name to go to the examination room, that is when I would go back inside”* (Adolescent mother 2)

*I used to hear the health workers talk,* [saying] *that I am too young to have a baby and I would feel embarrassed … ”*(Adolescent mother 5)

The younger (13–15 years) unmarried adolescents reported having faced more stigma (both self-stigma and from the community around them) for example inappropriate language from people, blames, rejection and isolation compared to the older (16–17 years) adolescents even if they were not married.

### Physical abuse and violation of human rights

A bout half of the adolescents also reported having faced physical violence, such as beatings, from parents but also partners who did not want to take responsibility for the pregnancy and sometimes also their parents, who disapproved of their pregnancy at such an early age. In some cases, the beatings led to hospitalization, and heavy virginal bleeding in two pregnant adolescents. Some (3 out of 5) married pregnant adolescents were denied food and bedding by their partners. Adolescents, who had been forced into marriage by the parents because they had conceived, expressed having faced more physical abuse compared to those who lived with their parents. Parents tended to be violent just at the start of the pregnancy among all the adolescents interviewed and later calmed down. However, majority of the pregnant adolescents who lived with their partners continued to be physically abused by their partners throughout the pregnancy and even after delivery.

*“There was a time he* [my partner] *beat me and my stomach started paining so much I could not eat and even lost my appetite … When* [my partner’s] *mother prepares food and serves me, he* [my partner] *would take my plate and pour it* [away] *saying that I should not eat their food; that I should go back to our home to my parents”* (Adolescent mother 1)

*“It is important to make her* [an adolescent] *know that she can also get support like any pregnant mother can get [support] because some parents become so harsh to their daughters reaching to levels of beating them and taking them to police. That is why other adolescents end up in problems like committing suicide or carrying out abortions; it is because their parents threaten them a lot so they end up making wrong decisions”* (Adolescent mother 3)

### Lack of legal and cultural protection

Our study found that in the community, men who kidnapped adolescent girls and forced them into sex, which led to pregnancies, were often not prosecuted or condemned by the community. When the adolescents got pregnant, cultural practices or unwritten community by-laws in the name of ‘fines’ paused a big challenge. Parents of the pregnant adolescents collected a ‘fine’ from the adolescents’ partners. Partners of the pregnant adolescents and their families often used the ‘fine’ as ‘bride price’ to claim the adolescent girls as their wives and these adolescent girls were often forced to marry their partners against their will.

*“After three days the brother of my partner came here and asked my mother to allow them to go with me. She told them that ‘get me my things* [‘fine’ in form of money] *and I will let her go.’ But they had come with only half of the money, so she told them that if you complete paying all the money, you can come and take her”. (Adolescent mother 10)*

These were mainly driven by unequal power relations between adolescents, their partners and parents in which case the adolescent had little or no say and decisions were taken on their behalf. Almost all (13/15) adolescents interviewed told us that their pregnancy cases were reported to police by their parents. However, even when cases were reported to police and should have been taken as defilement criminal cases for further legal intervention in court, the police were reported not to have been that helpful. In all the 13 cases, the police instead only arrested the pregnant adolescents and their partners and then told the parents of the adolescents and partners to negotiate. The negotiations were what often led to the fines that the partners of the adolescents paid to the parents of the adolescents as echoed below.

*“ … he* [my father] *came with the Security guys and we were arrested and taken to the police; from there he [my partner] was given a fine of 1,100,000/-UGX* [300USD]*. So, after paying that money, I was told to come back home and the boy also to go to his home and that is what we did but I was now pregnant. So, the boy came for me and took me to his place to stay with him. Now there was a balance of Shs.60,000*/-UGX [17USD] *which they brought and cleared”.* (Adolescent mother 11)

### Inadequate adolescent friendly services at health facilities

All the Adolescents interviewed faced challenges including; limited visual and auditory privacy, long waiting times, non-affordable maternal health services, limited family planning services, and lack of post-abortion care services. This made the seeking of health services an inconvenience especially for unmarried adolescents and led to shunning of health services. Although all the facilities where the adolescents sought maternal care were public facilities where services and commodities are supposed to be free, due to frequent lack of drugs and stock outs, adolescents were expected to purchase some medications and birth items, which were expensive for those without partner's or parent's support. These challenges are further expressed in the quotes below.

*“I think they should separate adolescents from the older mothers during ANC because of the age difference. We don't feel free when we sit together with them during ANC sessions. It is better if adolescents are managed separately”.* (Adolescent mother 12)

*“Yes, but they [the health staff] would shut it [the door] later after you have come out. You would be examined when the other mothers are seeing [you],then later the door would be closed”.* (Adolescent 10)

The inadequate adolescent friendly services were amplified by inflexible health facility regulation such as; the insistence that partners of pregnant women accompany them to the health facility when accessing maternal services, and attend health facilities in appropriate maternity wear. Health workers often gave priority to women who were escorted by their partners and in some cases, adolescent mothers were not attended to because they did not go with their partners for antenatal services since their partners had denied responsibility for the pregnancy. Most adolescents could not afford to buy loose fitting maternity wear thus wore their pre pregnancy clothes like old school uniform which were tight and uncomfortable and the health workers would not attend to them in such wear. Most partners of adolescents particularly the unmarried had denied responsibility for the pregnancies and so could neither escort them to the facility nor buy them maternity clothes yet health workers would not attend to adolescents who went to seek maternal health services without their partners.

*“ … Whenever they* [health workers] *could tell me that, in the next appointment, I should come with my partner. I would also go back and tell* him [my partner] *but he would just keep quiet not until the last time when I was now about to deliver when I told him that health workers say we should go together to the facility, he simply said I rather escort another woman for ANC but not you’ … ”* (Adolescent mother 1)

*“They would also ask me why am putting on a torn or tight dress? Still I would tell them [the health providers] that my partner does not buy me [the necessary clothing]. What they [the health providers] would do is to tell you to go back, we shall attend to you next time when you come in a maternity dress. So, one day I went back home without getting services … .”* (Adolescent mother 1)

### Lack of birth preparedness

Most adolescent mothers lacked birth-preparedness requirements and often struggled to have the items necessary for delivery. Some of the items that a woman is expected to have include; baby clothes, mama-kit, basin, soap and detergent and some money which can be used to fuel the ambulance to a referral hospital in case of any emergency. This is the case because the health facilities cannot afford these facilities for the mothers due to inadequate financing of the public health facilities.. Adolescents who did not have partner and family support even had greater challenges in birth preparedness. In some instances, the mothers of pregnant adolescents had to sell their personal items in order to provide birth-preparedness items for their pregnant daughters. Where the parents of the adolescent could not afford, the adolescents faced delivery head on without basic delivery items like basin, soap, and baby clothes.

*“My mother gave out her gomesi* [cloth] *to one of her friends who then gave her money to buy baby clothes and sheets. She* [my mother] *gave me money and told me to go and buy for myself 2 pairs of knickers and then baby clothes.* (Adolescent mother 14)

*“We did not even buy baby clothes because we did not have money … I did not have baby’s bathing soap, detergents for washing, baby powder and baby nappies. Up to now, I have not got some things like nappies and baby overalls. She* [my baby] *has only* one [overall] *, which she has now outgrown”*. (Adolescent mother 9)

### Extent to which the CSC addressed the maternal health challenges of adolescents

We assessed the extent to which the CSCs addressed the maternal health challenges of adolescents by exploring their attendance and participation during the meetings and the process of selection of indicators to be included in the CSC.

### Attendance of CSC meetings by the adolescents

Although adolescents were part of the target group for the meetings, very few adolescents attended the CSC meetings. Those who were in school were unable to attend because the meetings were held during school hours however, even those out of school did not attend in large numbers. Some adolescents also thought that the CSC meetings were meant for only adults to attend, as mobilization of people to attend the meetings did not emphatically mention their need to attend the CSC meeting.

*“Sometimes these meetings are organized during school days and so some of them take place while I am at school and I think the same thing affects other adolescents who are also still students and besides the real timing is during class time so when you return from school you just hear that there was a meeting somewhere”* (Adolescent 15)

### Participation of adolescents during the meetings

The few adolescents who attended the CSC meetings feared to openly air out their maternal health challenges in the presence of adults. Furthermore, they feared to speak about sexuality related issues in the presence of adults, as they could be labeled promiscuous. Hence the adolescents tended not to speak up about their specific maternal health challenges but rather went along with the opinions of the majority, which focused on issues that affect all women in the community as echoed below.

*“We* [adolescents] *were just quiet in the meeting* [CSC interface meeting] *… in that meeting we were with the mature women and men which scared us a lot. The truth is some of the adolescents who were in that meeting were fearing to talk … ”* (Adolescent 7)

### Selection of indicators to be included in the CSC

During the initial CSC meetings, the communities were asked to brainstorm on the main MNH service delivery and utilization issues that affected their communities. Then by show of hands and going by majority vote, they prioritized a few issues that they included on the CSC and scored on those going forward. Because the adolescents were few in the meetings, exacerbated by their fear to talk about sexual related issues in public, their issues never got to be mentioned and therefore did not get to the priority list for scoring and subsequent discussions. The common indicators selected and scored by the different sub counties as documented by Ekirapa-Kiracho et al. [41] included; mothers attending ANC in 1st trimester, men escorting partners for ANC, transport to health facility available, availability of midwives, availability of delivery beds, availability of drugs and Traditional Birth Attendant (TBA) deliveries. All the indicators did not specifically target the adolescent specific challenges but rather general maternal challenges. While some indicators such as men escorting their wives and partners for ANC could have to some extent addressed the psychosocial support needs of the adolescents, during the action planning the adolescent specific challenges related to partner acceptance were missed and therefore not addressed. This was reflected in the following quote.

*“nothing specifically attached to adolescent maternal health needs was discussed, they were just talking about the general things that take place in the health facility what the health workers should do and the community response but to say that they mention about the special needs of the adolescents; no”.* (Adolescent 3)

The general reflection from all the adolescents was that the CSC did not address any specific maternal health challenges faced by the adolescents. All the challenges such as psychosocial challenges, physical abuse and violation of human rights, lack of legal and cultural protection, inadequate adolescent-friendly services at health facilities, birth preparedness were not included in the CSC to specifically address adolescent challenges.

#### Study limitation

**We did not consider a** conceptual theory for this study as is recommended for more rigor and generalizability, however we think that our findings are relevant and can be useful for CSC projects and similar more rigorous studies that will included conceptual theories are recommended.

## Discussion

This study found that the adolescent specific maternal health challenges included; Psychosocial challenges, Physical abuse and violation of human rights, lack of legal and cultural protection, inadequate adolescent friendly services at health facilities, and lack of birth preparedness. We also found that the Kibuku CSC did not specifically address these adolescent specific maternal health challenges. We discuss the challenges and the extent to which these were addressed in detail.

### Psychosocial challenges

Some of the adolescents were rejected by their parents and chased away from home mainly because their parents did not approve of their pregnancies at early ages and also didn't want to take responsibility of caring for the pregnant adolescent. This forced some adolescents into marriage so that the partner responsible for the pregnancy would take care of them as also observed in other places in Uganda [42]. Some partners denied responsibility for the pregnancy due to fear of the financial and social responsibilities [43] and thus asked the adolescents to abort. This combination of lack of family, partner and community support for young pregnant mothers reinforcing their exclusion from maternal health services is a widespread phenomenon with similar experiences being reported in other parts of Uganda [8, 23, 42, 44, 45]. During the CSC community scoring meetings, there would have been a possibility of voicing these psychosocial challenges experienced by pregnant adolescents so that then the communities would deliberate on them and find joint solutions however this was not the case just as it was with the rest of the other challenges.

### Adolescent friendly services

The adolescents interviewed in our study expressed inadequacies in Adolescent-friendly services yet these are very important in enabling adolescents to seek maternal health services such as antenatal care (ANC) and health facility delivery [46]. An Asian study found that an increase in adolescent-friendly services was accompanied by improvements in the health-seeking behaviors of adolescents for maternal health services [47]. In Uganda there is a clear need to have flexible regulations and norms of maternal health service delivery in order to better accommodate adolescents [8, 23, 42]. Most adolescents’ partners deny their pregnancies, ran away for fear of imprisonment for impregnating an adolescent and the implications for financial responsibility, or feared to be seen seeking ANC with a young girl. Health workers often fail to recognize these vulnerabilities and sometimes deny services to adolescents who don’t conform to these requirements, even though they had minimal effects on the girls’ health and immediate pregnancy needs. The CSC dialogues would have created a platform for the health workers to understand the challenges faced by the adolescents who get pregnant so that they would be flexible when handling the adolescents. From the recommendations of the dialogues would have probably come recommendations like the need to train health workers on treating adolescents with dignity and respect, on sexual reproductive rights, and on how to counsel adolescents [27, 47–49] so that they get flexibility while working with adolescents.

Many adolescents and their caregivers, in Uganda and in other sub-Saharan countries, do not have adequate information on adolescent maternal health needs, ANC [47, 50] and sex education [51, 52]. The World Health Organization therefore recommends the need to increase information flow among adolescents [53–55]. Policies targeting adolescents in school to inform them about adolescent maternal health needs should be put in place [56, 57] and service providers and policy makers should engage adolescents in decisions pertaining to their health and needs [58].

### Birth preparedness

Birth preparedness in this context refers to a pregnant mother having the required birth items by health workers to help her during delivery for example; baby clothes, mama-kit, basin, soap and detergent and some money which can be used to fuel the ambulance to a referral hospital in case of emergency. Birth preparedness is fundamental in enabling pregnant women to deliver at health facilities [59]. However, adolescents are often times not prepared for birth because they do not have the financial capacity to purchase necessary items [59, 60]. Majority of the adolescents didn’t have a source of income since they got pregnant while still at school. A similar study in Nepal found that younger women were less prepared for birth [61]. Adolescents who do not have their partners’ support [48, 62] and families’ support [51] and whose family members are not able or willing to support are financially and socially disempowered. Thus there is need to strengthen male involvement through appropriate policies that recognize adolescent boys’ and men’s roles in pregnancy and find ways to support male partners’ involvement in MNH services as male support is key in provision for birth preparedness [63]. The involvement of community health workers and other community leaders can also help improve birth preparedness [64, 65].

### Cultural and legal protection

This study found that adolescent mothers are sexually and physically abused leading to pregnancies [66] and some Western studies found similar results [67]. This may be because male adolescents are more hostile and physically violent compared to females [68] but also due to patriarchy and the devaluing of women’s roles and stereotypical gender norms which state that girls should not get pregnant, and blame girls for doing so. Thus, adolescent mothers need legal and cultural protection because they are not empowered enough to defend themselves from their partners and other family members. Similar results showed that the lack of social family security systems expose adolescents to violence and sexual assault [69–71] yet where family and relatives offer social support and protection to the pregnant adolescents, the adolescents are better empowered [72]. Adolescents also need to be protected from parents and guardians who want to take advantage of their pregnancies for financial gains in form of ‘fines’. These fines are a form of abatement of crime and could amount to prosecution in the courts of Law. Adolescents are not well informed about the law on defilement and they are not included in any decision-making about their and their babies’ futures [73] and this further dis-empowers them.

### Extent to which the CSC addressed the maternal health challenges of adolescents

The Kibuku CSC did not specifically address any of the discussed maternal health challenges faced by adolescents but rather addressed general maternal health issues affecting the community as a whole. Special needs of adolescents got left out in favor of the general community needs since no one voiced the needs of adolescents strongly enough. Although several of the general indicators such as availability of transport to the health facility, delivery with traditional birth attendants, men escorting their partners could have addressed their needs, this did not happen. Strategies to address adolescent specific concerns were not identified since no one emphasized their needs as a special category. For example whereas during the CSC meetings men were asked to escort their wives and sensitization was done to encourage the men to do so, sensitization about the need to support pregnant adolescents whose partners and parents often rejected was not identified, hence they were not included in the work plans and subsequently their challenges were not addressed.

Very few adolescents attended the CSC meetings because they viewed it as a community meeting of the adults. There was no deliberate effort to specifically reach out and mobilize the adolescents for the CSC meetings. Often times the meetings were convened during school time and the adolescents had to choose between classes and the meeting and school attendance was preferred. During the CSC meetings, even the few adolescents who attended hardly participated to raise their maternal health challenges because of fear to speak amidst many adults about issues concerning their sexuality. A study conducted in DR Congo found that adolescents fear to freely express themselves in public [74]. This made it hard for the CSC as a social accountability tool to address the adolescent specific challenges since they did not have their own meetings where they would have felt safe enough to voice their problems. A study in Ethiopia recommended the need to design audience- specific strategies in order to enable adolescents freely express their sexual concerns [75]. Failure to address the adolescent related challenges in the CSC could also have been a result of limited community awareness about the challenges faced by the adolescents. An evaluation of eight projects on CSC mostly on health in five African countries also reported that the projects found challenges, in the inclusion of marginalized populations in the processes of the CSC [37]. A number of social accountability projects and evaluations in Asia and Africa have not included marginalized community groups especially in maternal health like the adolescents in their interventions [76–78]. Some studies are in agreement with our thinking that social accountability projects also need to specifically pay attention to marginalized groups like adolescents in the community [77, 79].

The CSC processes need to look at innovative ways to bring the voices of the adolescents into their discussions for example through separate adolescent meetings before the main community meetings from which adolescent voices can be collected then presented by a representative. Where health facility committees are used as social accountability platforms as was the case in Malawi [78], these committees would consider including adolescent representatives who collect and present the voices of the adolescents in the different forums. It is also possible to deliberately prompt the initial meetings on social accountability to think and talk about the maternal health challenges faced by adolescents during the selection of indicators for improvement. Once these adolescent challenges have been included in the score card at the start, the project is then sure of their subsequent consideration for scoring and improvement.

## Conclusions

The maternal health challenges faced by adolescents in Kibuku have a cultural, legal, social and health service dimension. There is therefore need to look at a multi-faceted approach to holistically look at community behavior, strengthening of implementation of laws on defilement and policies on adolescent friendly health services. There is also a need for studies and interventions that can target and empower adolescents with information and other mechanisms to strengthen their ability to voice the maternal health issues affecting them. Long built cultural constructions and norms which entrench practices such as forced early marriage need to be tackled through intersectoral dialogues not only health domain.

Based on the evidence gathered in this paper, the CSC in Kibuku district did not address the specific maternal health challenges of adolescents. In order for CSCs to address the maternal health challenges of adolescents, there is need to have specific meetings for the adolescents. In addition, there should be deliberate inclusion of indicators that address their needs, as well as a deliberate attempt to ensure that these needs are addressed during the action planning and implementation phases.

## Data Availability

The datasets used and/or analyzed during the current study are available from the corresponding author on reasonable request.

## Abbreviations

ANC: Antenatal care
CSC: Community score card
IDI: In-depth Interview
LMICs: Low and middle income countries
MakSPH HDREC: Makerere University School of Public Health Higher Degrees Research and Ethics Committee
MNH: Maternal and newborn health
SRH: Sexual and reproductive health
TBA: Traditional birth attendant
UNCST: Uganda National Council of Science and Technology
VHTs: Village Health Teams
